# Horizontal gaze palsy and progressive scoliosis: magnetic resonance imaging features and surgical treatment

**DOI:** 10.1590/S1679-45082017AI3969

**Published:** 2017

**Authors:** Benedito Jamilson Araújo Pereira, Ulysses Caus Batista, Fúlvio Nicolau Bechelli, Carlos Alberto Afonso Ribeiro, Carlos Vanderlei Medeiros de Holanda, Paulo Eduardo Carvalho Galvão

**Affiliations:** 1 Hospital Beneficência Portuguesa, São Paulo, SP, Brasil.; 2 Universidade Estadual de Campinas, Campinas, SP, Brasil.

**Figure 1:**
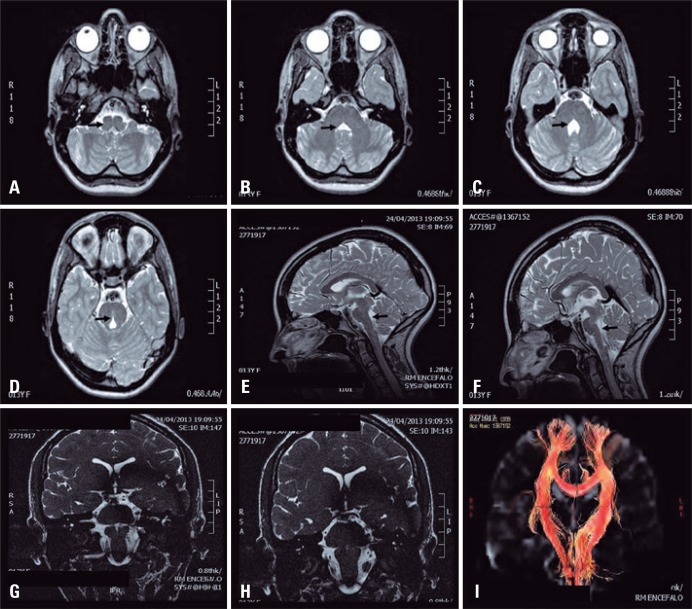
Pre-operative images. (A to D) Magnetic resonance imaging axial T2 shows the hypoplastic pons and medulla with a prominent midline cleft. (E and F) Magnetic resonance imaging sagittal T2 of the brain shows depression of the floor of the fourth ventricle. The pons and medulla oblongata have a reduced volume. (G and H) Magnetic resonance imaging coronal T2. (I) Tractography showed no crossing over of major fibers at the level of pons

**Figure 2:**
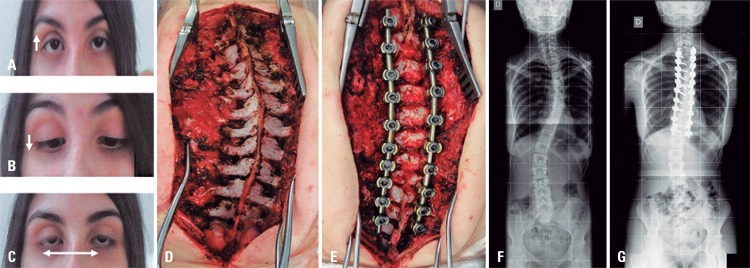
Operative findings. (A and B) Vertical eye movements within normal limits. (C) Absence of surgical features of horizontal eye movements. (D) Exposure of thoracic spine scoliosis with left convexity. (E) Posterior spinal fusion from the second to the eleventh thoracic level. (F) The anteroposterior radiograph before surgery. (G) The anteroposterior radiograph after surgery, showed degree of coronal balance and correction obtained

A 13-year-old patient was referred for surgical treatment of scoliosis. Her neurological examination revealed the complete loss of horizontal eye movements with normal vertical gaze, suggesting diagnosis of horizontal gaze palsy and progressive scoliosis. The magnetic resonance imaging revealed a hypoplastic pons and medulla with a prominent midline cleft, therefore resulting in characteristic “butterfly” configuration.^(1)^ The tractography showed absence of major crossing pathways within the pons and midbrain, and normal interhemispheric connections in the corpus callosum (Figure 1). Surgical treatment for scoliosis was performed successfully (Figure 2).

Horizontal gaze palsy with progressive scoliosis is a rare autosomal recessive disorder characterized by congenital absence of normal horizontal eye movements and progressive scoliosis during childhood and adolescence. Heterozygous mutations in ROBO3 are reported as a cause of this disorder.^(2)^
